# Cardiotoxicity in breast cancer patients treated with chemoradiotherapy

**DOI:** 10.37349/etat.2026.1002401

**Published:** 2026-09-14

**Authors:** Peter Emerson, Liza Thomas

**Affiliations:** N.N. Petrov Institute of Oncology, Russia; ^1^Department of Cardiology, Westmead Hospital, Westmead 2145, Australia; ^2^Westmead Clinical School, The University of Sydney, Camperdown 2050, Australia

**Keywords:** breast cancer, anthracyclines, cardio-oncology, strain

## Abstract

With national breast cancer screening programs leading to earlier diagnosis and improved treatments, more women are achieving long-term remission. However, this has resulted in rising rates of cardiotoxicity in breast cancer survivors consequent to their cancer therapies. Anthracyclines, a cornerstone treatment in breast cancer, carry a significant risk of short- and long-term cardiotoxicity through inhibition of topoisomerase II, preventing DNA repair and increasing oxidative stress. This most commonly manifests in the form of left ventricular (LV) systolic dysfunction or heart failure, and in severe cases, death. Many of the other treatments, including taxanes, cyclophosphamide, human epidermal growth factor receptor 2 inhibitors, immunotherapies, hormone therapies, and radiotherapy, also carry a risk of cardiac injury in the form of heart failure, arrhythmias, myocarditis, and pericardial disease. Surveillance has traditionally been performed by monitoring LV ejection fraction by transthoracic echocardiography. However, this has been found to be a late marker of cardiotoxicity, with damage potentially irreversible, and may reflect a missed opportunity for early intervention. Research has focused on identifying cardiac biomarkers and advanced imaging techniques, including strain analysis, to detect subclinical myocardial injury or dysfunction prior to irreversible damage, with LV global longitudinal strain a sensitive marker of subclinical LV systolic dysfunction and superior predictor of future cardiovascular events. Further research is needed to establish standardized guidelines on surveillance and management of this vulnerable patient cohort.

## Introduction

Breast cancer is one of the biggest global health challenges we face, as the most common cancer diagnosed in women [1]. Its incidence has continued to rise, with global age-standardized incidence rates estimated at 48 per 100,000 women [2]. This has likely been consequent in part to improved detection with screening programs as well as improved community and individual awareness of early symptoms and signs [3]. However, exposure to hormone therapy, rising obesity levels, increased alcohol intake and increasing nulliparity have likely additionally contributed to its increased incidence, with cases worldwide expected to exceed 6 million annually by 2050 [2]. However, with the advent of newer anti-cancer agents and earlier diagnosis from national screening programs, mortality rates have drastically declined by over 44%, with 5-year survival rates now exceeding 90% when diagnosed early [4, 5].

However, with improved remission rates, long-term cardiotoxicity consequent to cancer treatments has surfaced as a new global health challenge affecting a significant proportion of breast-cancer survivors each year, with studies demonstrating over 15% of women treated with chemotherapy or targeted therapy developing therapy-related cardiotoxicity [6]. At 10-year follow-up, cardiac dysfunction remains the leading cause of mortality amongst breast cancer survivors, exceeding that of cancer recurrence [6, 7]. With these emerging data, there has been a shift in focus from solely maintenance of remission to prevention and management of the long-term cardiac effects of treatment in breast cancer survivors. This has led to a field of expertise involving close collaboration between Oncologists and Cardiologists termed Cardio-oncology, focused on the prevention and management of cardiovascular complications in this vulnerable patient cohort.

## Breast cancer management

Management of breast cancer, depending on the stage and progression of disease, typically involves some combination of surgical resection, systemic treatments including chemotherapy, hormone therapy, immunotherapy and targeted therapy as well as radiation therapy [1]. In management plans with curative intentions, surgery is typically performed as lumpectomy or mastectomy with possible axillary lymph node dissection, depending on the size and spread of the tumour [8]. However, with improved understanding in achieving long-term cancer remission, even those patients with early-stage breast cancer are increasingly treated with systemic therapies and radiotherapy [9].

### Chemotherapy

Systemic therapies in breast cancer treatment can be broadly categorized into chemotherapy, targeted therapies, immunotherapies, as well as endocrine (hormone) therapies. Anthracyclines have long been the cornerstone treatment in breast cancer for decades [10]. Discovered in the 1940s, Daunorubicin was isolated from the bacterium *Streptomyces* and demonstrated to have clinical utility in the treatment of acute leukaemia. Doxorubicin, a precursor, was not long after discovered, with demonstrated utility beyond leukaemia in the treatment of solid organ tumours including breast cancer. Decades later, even with accelerated advancements and development of novel anti-cancer agents, anthracyclines remain one of, if not the most effective agents in the treatment of breast cancer and numerous other malignancies including acute leukaemia, bladder cancer, lung cancer and various others [11, 12]. Though these agents have been used for decades, their exact mechanism of action is not completely understood. One of the most widely accepted theories is that anthracyclines interact with topoisomerase II, an enzyme integral to DNA metabolism [13]. By binding to topoisomerase II, it prevents re-ligation of the double-stranded DNA breaks, thus inhibiting cell growth and promoting apoptosis. Other theories include excess oxidative stress from increased reactive oxygen species (ROS), DNA intercalation inhibiting DNA and RNA synthesis, and blockade of transcription factors that inhibit cell cycle progression.

Taxanes are another class of chemotherapeutic agents commonly used in the treatment of breast cancer. Paclitaxel was the first agent from this class that was approved for treatment of refractory ovarian cancer in the 1990s [14]. Since then, it is now widely utilized in various malignancies including but not limited to breast cancer, non-small cell lung cancer and prostate cancer. They primarily work through suppression of microtubule function, inhibiting a variety of processes including cell signalling, migration and division, eventually inducing cell death [15]. Anthracyclines in combination with taxanes are a common chemotherapy regimen for treatment of high-risk early breast cancer [16].

Cyclophosphamide is an alkylating agent that was derived from research into mustard gas used during World War II [17]. The metabolites of cyclophosphamide create cross-links between DNA strands, inhibiting protein synthesis and resulting in cellular apoptosis [18]. It is used in the treatment primarily of haematological malignancies as well as an immunosuppressive agent in several autoimmune conditions/organ transplants. In breast cancer, it represents an alternative agent for those patients at lower risk of spread, or for whom other agents are contraindicated [19].

### Targeted therapies

Human epidermal growth factor receptor 2 (HER2) is a transmembrane protein belonging to the epidermal growth factor receptor (EGFR) family of tyrosine kinases [20]. It has been demonstrated to be overexpressed in a number of malignancies, including 20% of all breast cancer cases, as well as in a significant proportion of ovarian, gastric, and colorectal cancers, and has been associated with a poorer prognosis. Trastuzumab, a monoclonal antibody and the first HER2 inhibitor that entered clinical practice in the late 1990s, revolutionized treatment of HER2-positive breast cancers as a targeted therapy through binding to the specific receptors and preventing signal transduction, eventually inducing cell death [21]. Newer HER2 inhibitors have been engineered and significantly improved survival rates in patients with HER2-positive breast cancer.

### Immunotherapies

Beyond this, there have also been newer therapies developed to target different molecules on the cancer cells. These include immunotherapy drugs that boost the patient’s immune system to remove cancer cells, such as the programmed cell death protein 1 (PD-1) and programmed cell death ligand 1 (PD-L1) inhibitors that are often used in triple-negative breast cancer [22, 23]. Pembrolizumab, a PD-1 inhibitor, is widely used in early-stage triple-negative breast cancer as well as those with metastatic, PD-L1 positive disease [24]. By binding to PD-1 on immune T cells, it prevents the binding of PD-L1 on cancer cells to PD-1, allowing the T cells to recognise and kill the cancer cells.

### Endocrine therapy

Since the late 19th century, there has been a growing understanding of the influence of the ovaries on breast cancer progression [25]. Though initially oophorectomy was performed for premenopausal women with breast cancer, growing research has demonstrated the prevalence of estrogen receptor (ER) and progesterone receptor (PR) in malignant cells. 80% of breast cancers are ER-positive, and of these 65% are also PR-positive; thus, most women with breast cancer are treated with endocrine therapy [26]. Tamoxifen is a selective ER modulator that acts as both an agonist and antagonist on ERs. In breast tissue, it competes with endogenous estrogen for binding and exerts antiestrogenic effects, inhibiting cell cycling of malignant cells. However, in other tissues such as bone, it stimulates estrogenic effects in regulating bone metabolism [27]. In post-menopausal women, aromatase inhibitors have shown superiority to tamoxifen by inhibiting the enzyme aromatase, thereby preventing the conversion of androgens to estrogen in peripheral tissues such as the adrenals, skin and adipose tissue (which are the primary source of estrogen post-menopause) [28].

### Radiation therapy

Radiation treatment represents the only other form of localised therapy along with surgery in the treatment of breast cancer. It aims to damage the DNA in cancer cells (though inadvertently in surrounding healthy cells as well) through ionising radiation that promotes cell death [29]. It can either be given to the entire breast tissue or to part of it in patients at low risk of spread [30]. It is widely used in breast cancer (as well as in several other cancers) and has helped achieve higher cure rates in those without metastatic spread, with over 50% of breast cancer patients now receiving radiotherapy sometime during the course of their treatment [31].

## Cancer therapy-related cardiotoxicity

**Figure 1:**
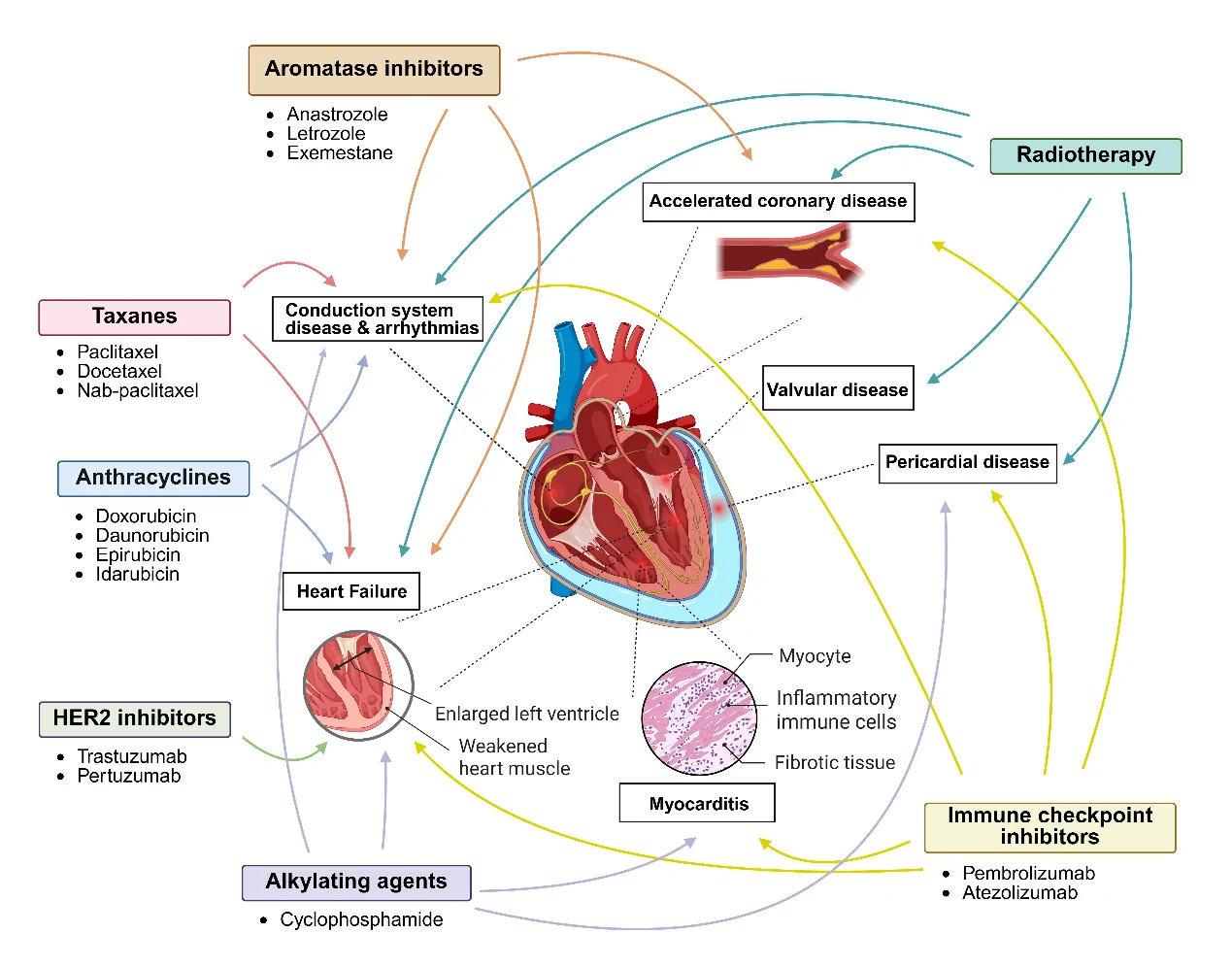
**Clinical manifestations of cardiotoxicity with systemic therapies and radiotherapy used in breast cancer.** Clinical manifestations of cardiotoxicity in cancer patients treated with systemic therapies and radiotherapy include heart failure (most common), arrhythmias/conduction disease including QT prolongation or sinus node/atrioventricular node dysfunction, coronary artery disease, valvular dysfunction, myocarditis, or pericarditis. Created in BioRender. Thomas, L. (2026) https://BioRender.com/5pv46t0.

With the advent of numerous new breast cancer treatments, more patients are achieving long-term remission. However, with this, many patients are now facing the long-term adverse effects of the treatments received, particularly on the heart [32]. Cardiotoxicity from breast cancer therapy has emerged as a leading cause of non-cancer-related morbidity and mortality in these patients. Studies have demonstrated that over 15% of breast cancer patients show signs of long-term cardiotoxicity, primarily in the form of reduction in left ventricular ejection fraction (LVEF) [6]. However, this is likely underestimated, with LVEF considered a late marker of cardiac damage, nor does it account for other forms of cardiotoxicity, including diastolic dysfunction, cardiac arrhythmias, myocardial ischaemia or pericardial disease (Figure 1) [33]. In fact, a greater percentage of patients presenting with heart failure following breast cancer treatment present with heart failure with preserved ejection fraction (HFpEF), compared to heart failure with reduced ejection fraction (HFrEF), with those patients with HFpEF often suffering a poorer prognosis [34].

The mechanism of cardiotoxicity in these patients can vary significantly and often depends on the treatments received (Table 1). Anthracyclines are among the most widely used treatments in breast cancer, though they are limited in their application by their cardiotoxic effects, with rates reported as high as 29% [10, 35]. Although their specific cardiotoxic mechanism remains unclear, several possible pathways have been identified through topoisomerase II inhibition, increased oxidative stress or mitochondrial dysfunction resulting in cardiomyocyte injury and death, as mentioned previously [36]. The cardiotoxic changes can present with overt clinical manifestations in the form of heart failure (most common), asymptomatic left ventricular (LV) dysfunction, or arrhythmia. These changes are potentially irreversible given the limited regenerative capacity of cardiomyocytes in adulthood [37, 38]. To mitigate these effects, treatment regimens have focused on reducing lifetime cumulative doses given its dose-dependent toxicity, extending infusion durations, using a liposomal formulation, and adding dexrazoxane (iron-chelating agent [39]. Though cardiotoxicity typically presents within two years of chemotherapy completion, late-onset cardiotoxicity has been reported beyond 7 years post anthracycline administration, necessitating long-term echocardiographic surveillance of these patients [38].

**Table 1:** Cardiotoxicity of systemic therapies and radiotherapy in breast cancer treatment.

**Category**	**Pathophysiology**	**Clinical Manifestations**	**Cardiotoxicity rates**
Anthracyclines	Topoisomerase II inhibition Increased ROS Mitochondrial dysfunction	Heart failure Arrhythmias	29% [35]
Taxanes	Massive histamine release	Conduction system disease Heart failure	5–20% [40]
Alkylating agents	Metabolite-induced increased ROS	Heart failure Arrhythmias Hemorrhagic myocarditis (severe) Pericarditis	Up to 28% [40]
HER2 inhibitors	HER2 inhibition reduces cardiomyocyte repair & survival	Heart failure	14% [41]
Immune checkpoint inhibitors	T-cell mediated inflammation	Myocarditis Pericarditis Takotsubo cardiomyopathy Coronary artery disease	3% [42]
Aromatase inhibitors	Estrogen-inhibition Reduced vasodilation and lipid metabolism	Heart failure Coronary artery disease Arrhythmias	13% [43]
Radiation therapy	Oxidative stress Endothelial injury Fibrosis	Coronary artery disease Constrictive pericarditis Heart failure Valvular dysfunction Arrhythmias	4%/Gy [44]

ROS: reactive oxygen species.

Cardiotoxicity rates in patients treated with taxanes range from 5–20% [40]. These patients primarily present with disorders of the conduction system, including QT prolongation, severe bradycardia, and atrial fibrillation [45]. This is thought to arise from a massive histamine release resulting in interruptions in cardiac conduction, predisposing patients to potentially fatal arrhythmias. It has also been associated with the development of heart failure, likely through increased ROS production and disruption of mitochondrial function and cardiomyocyte death. Though anthracycline-taxane combination regimens have demonstrated superior efficacy in achieving breast cancer remission, their concomitant use compounds the risk of heart failure, limiting their utility in many patients [45, 46].

Though cyclophosphamide is known to carry significant risks to the bladder and bone marrow, it also carries a risk of dose-dependent cardiotoxicity, with rates reported as high as 28% in intensive regimens [18, 40]. Its effects on the heart are thought to arise from its metabolites, resulting in oxidative stress and endothelial injury [47]. With this therapy, patients can present with heart failure, arrhythmias, or, in more severe cases, haemorrhagic myocarditis and pericardial effusion causing tamponade (Figure 1).

HER2 inhibitors report rates of cardiotoxicity as high as 14% when used in isolation, with even higher rates reported in patients previously treated or when concurrently receiving anthracyclines [41, 48]. Its mechanism is thought to be different to that of anthracycline-induced cardiotoxicity, without any ultrastructural changes noted in the myocardium [49]. It is thought to occur from direct inhibition of HER2 in cardiomyocytes, which are integral in maintaining cellular survival and repair. This increases the cells’ susceptibility to damage from increased oxidative stress, including in those receiving concomitant anthracycline. Hence, concomitant administration has been changed to sequential therapy following anthracyclines [50]. Patients typically present with symptomatic heart failure or asymptomatic left ventricular dysfunction. However, as opposed to anthracycline-induced cardiotoxicity, which can be difficult to reverse, cardiac dysfunction from HER2 inhibitors is often reversible with cessation of treatment.

Immune checkpoint inhibitors such as pembrolizumab have been used in high-risk early triple-negative breast cancer and in advanced disease where PD-1 is expressed on tumour cells [51]. By upregulating the body’s immune system to better remove cancer cells, these drugs inadvertently increase the risk of a plethora of immune-related adverse events in most organs, including the heart [52]. Through inhibition of PD-1/PD-L1, T cells mistakenly recognise cardiac cells as foreign pathogens and, through the release of inflammatory cytokines, can cause T-cell-mediated myocarditis [53]. Furthermore, PD-L1 has known cardioprotective effects by inflammatory suppression and cardioprotective signalling during ischaemia and infarction. Inhibition of PD-L1 can accelerate decompensation of pre-existing heart disease such as heart failure, arrhythmia or infarction in breast cancer patients treated with these medications. Studies have demonstrated a 3.1% incidence of cardiotoxicity in patients treated with these immunotherapies, with most patients presenting with myocarditis, pericarditis, arrhythmias or heart failure [42]. Though the rate of cardiotoxicity is relatively low in comparison to other breast cancer treatments, these patients present very unwell with severe and life-threatening cardiac complications, with rates of mortality with myocarditis as high as 50% [54].

While aromatase inhibitors have helped achieve strong long-term remission rates in hormone receptor-positive breast cancer, they have been associated with increased cardiovascular events [55]. As opposed to tamoxifen, which inhibits ERs on cancer cells, aromatase inhibitors reduce serum estrogen levels by over 95% in most individuals [56]. Estrogen plays an important role in lipid metabolism and vasodilation, which likely contribute to a degree of cardioprotection [57]. Several studies have demonstrated an increased incidence of cardiovascular events with the use of aromatase inhibitors, namely heart failure, myocardial infarction, arrhythmia and cardiovascular mortality [55]. This has limited their utility despite their strong efficacy in tumour suppression, with patients requiring extended monitoring of their cardiovascular and metabolic risk factors on treatment.

**Figure 2:**
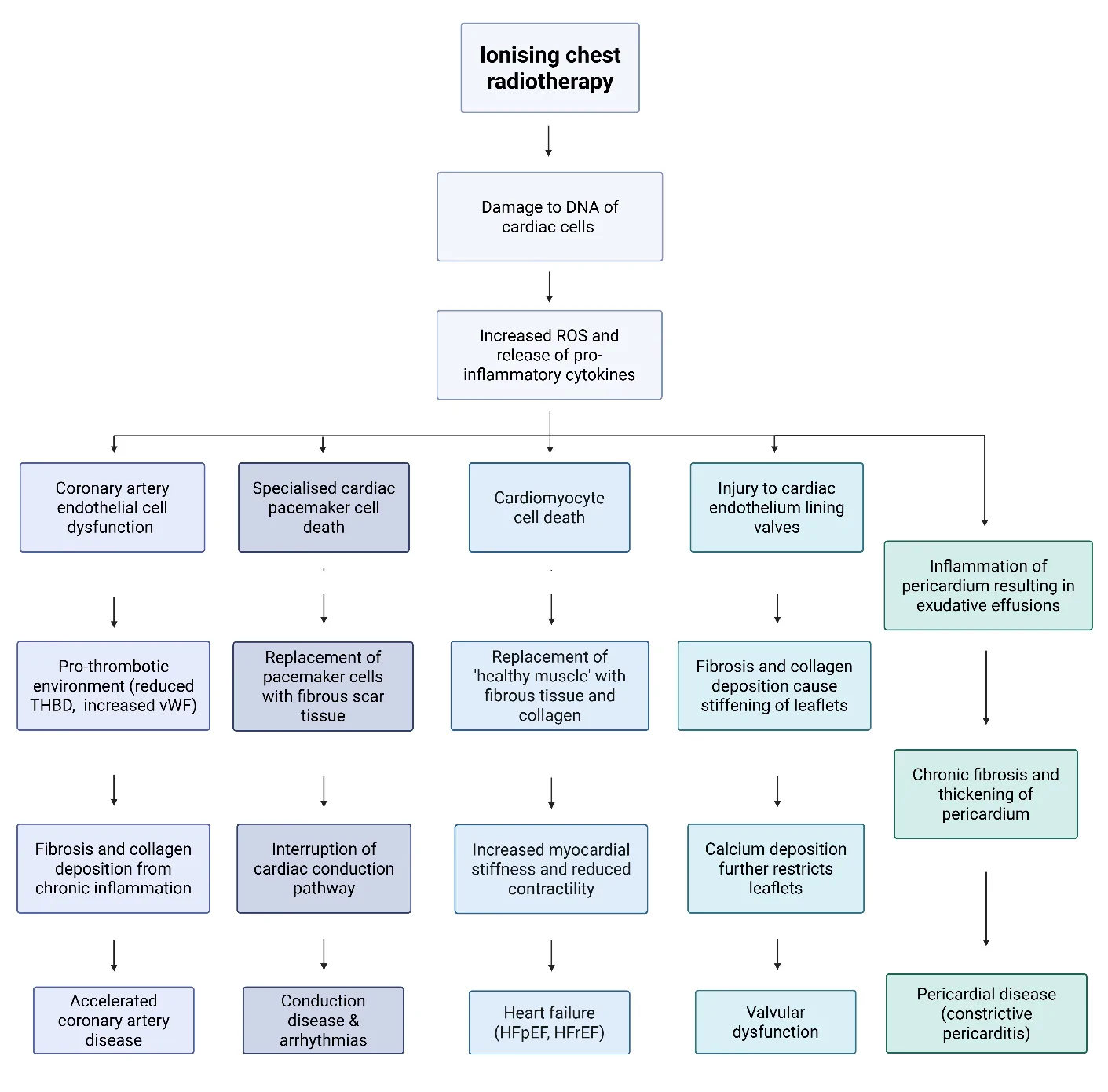
**Mechanisms of cardiotoxicity with chest radiotherapy.** Cardiotoxicity from ionising radiotherapy to the left side of the chest can manifest as accelerated coronary artery disease, conduction system abnormalities, heart failure, valvular dysfunction, or pericardial disease. HFpEF: heart failure with preserved ejection fraction; HFrEF: heart failure with reduced ejection fraction; ROS: reactive oxygen species; THBD: thrombomodulin; vWF: von Willebrand factor. Created in BioRender. Thomas, L. (2026) https://BioRender.com/sjq9yqp.

With most breast cancer patients receiving radiotherapy at some point during their treatment, many suffer from its short- and long-term effects on the heart [31]. Studies have shown that for each Gy increase in mean heart dose, the incidence of cardiotoxicity increases by 4% (with the average dose in left-sided breast cancer 3.6 Gy) [44, 58]. It can manifest in a number of ways, including accelerated coronary artery disease, pericardial disease, reduced left ventricular systolic function or symptomatic heart failure, valvular dysfunction, or conduction abnormalities, with higher rates reported with concurrent anthracycline use, the volume of heart irradiated, and those with pre-existing cardiovascular risk factors (Figure 2) [59–61]. This is thought to occur from the inflammatory, oxidative, and fibrotic effects on heart tissue exposed to ionising radiation, for which it is particularly susceptible given its limited regenerative capacity [37].

## Surveillance of cardiotoxicity

**Figure 3:**
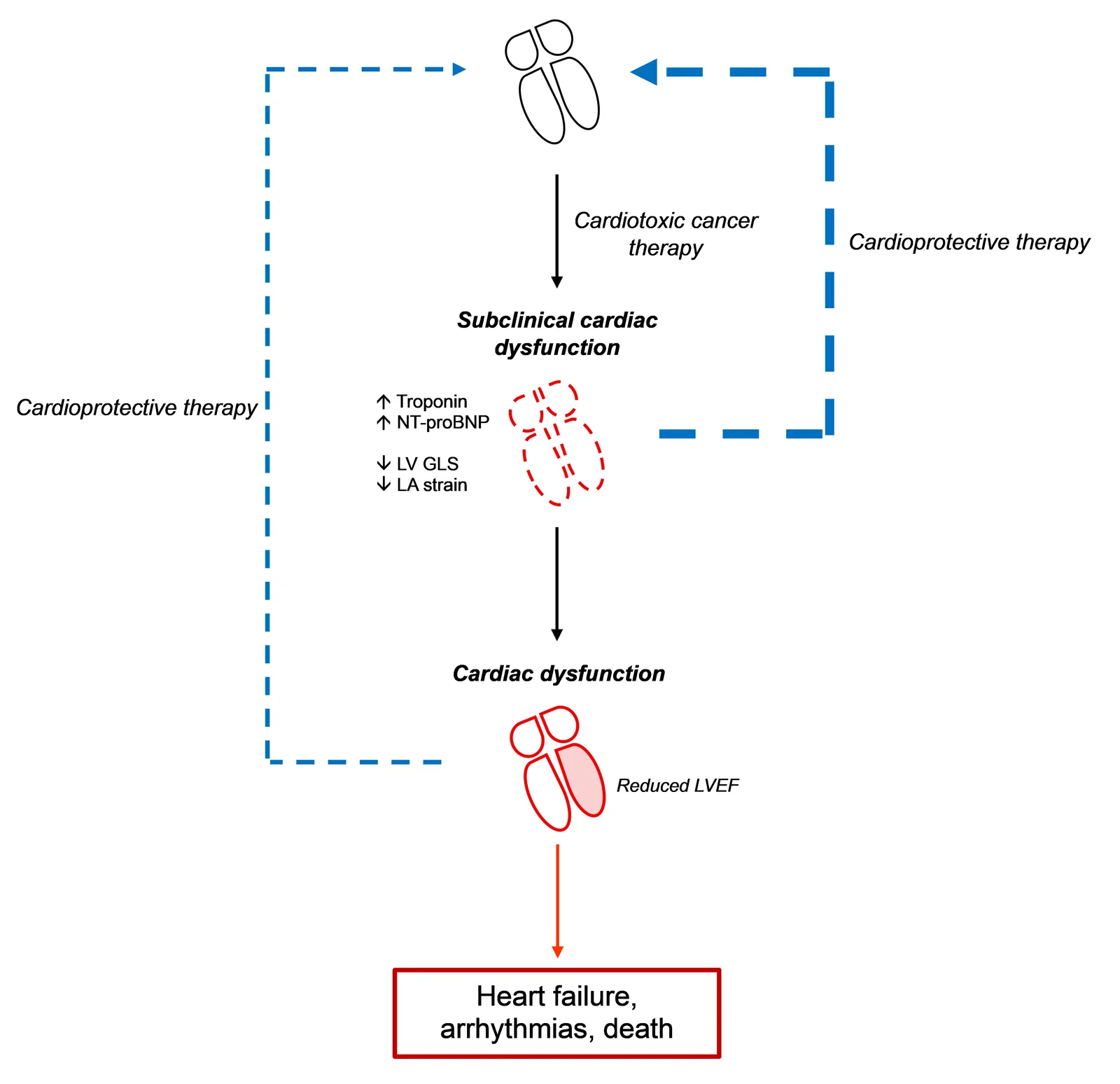
**Surveillance of cardiac dysfunction in cancer patients treated with cancer-related cardiotoxic therapy.** Through early identification of subclinical cardiac injury or dysfunction, more patients will respond to initiation of cardioprotective therapy prior to irreversible myocardial damage compared to traditional surveillance strategies using LVEF. LA: left atrial; LVEF: left ventricular ejection fraction; LV GLS: left ventricular global longitudinal strain; NT-proBNP: *N*-terminal pro-B-type natriuretic peptide.

With many of the treatments for breast cancer carrying significant risks of cardiotoxicity, particularly when used in combination, there has been a greater emphasis on early detection of cardiotoxicity in breast cancer patients. This is with the objective that treatment cessation or early intervention with heart failure or anti-arrhythmic therapy can reverse the damage and prevent adverse cardiovascular events (Figure 3). This has primarily been investigated through surveillance using cardiac biomarkers or imaging modalities.

### Cardiac biomarkers

One method of cardiotoxicity surveillance has been through monitoring of cardiac biomarkers before, during, and after treatment [62]. The most commonly used markers are cardiac-specific troponins (cTnT, cTnI) and natriuretic peptides. Cardiac troponins are regulatory proteins found predominantly, or in the case of troponin I exclusively, in the heart that assist in the interaction between actin and myosin to facilitate coordinated contraction [63]. As cardiomyocytes are irreversibly damaged, they release troponins into the systemic circulation, making them a sensitive and specific marker of myocardial injury [64]. They are the gold-standard of diagnosis of myocardial injury in acute coronary syndromes, including myocardial infarction.

Natriuretic peptides are short-chain proteins from the heart that function as hormones that regulate cardiovascular homeostasis through their vasodilatory, natriuretic, and diuretic effects [65]. There are three key types: atrial natriuretic peptide (ANP), b-type (formerly brain) natriuretic peptide (BNP), and c-type natriuretic peptide (CNP), produced by atrial cardiomyocytes, ventricular cardiomyocytes, and endothelial cells, respectively [62]. Among these, BNP is typically preferred as a marker given its reflection of ventricular pressure and load. When ventricular cardiomyocytes are stimulated to produce biologically active BNP from its precursor proBNP, *N*-terminal pro-B-type natriuretic peptide (NT-proBNP, a biologically inactive peptide) is released into the systemic circulation in equal amounts [66]. NT-proBNP is the preferred marker over BNP given it is a more stable compound, has fewer interactions with cardiac medications, and levels do not fluctuate with the circadian rhythm [62].

Cardiac biomarkers represent a sensitive marker of early cardiotoxicity given the changes in serum concentration in subclinical injury [67]. In addition, there have been studies that have looked at their utility in risk stratification for the development of cancer therapy-related cardiac dysfunction (CTRCD), which can alter an Oncologist’s choice of treatment regimen [68]. In addition to this, changes in troponin and NT-proBNP have been shown to correlate with the degree of LV systolic dysfunction. Beyond surveillance of cardiotoxicity, they also provide an assessment of the degree of cardiac dysfunction [68]. While troponin and NT-proBNP represent sensitive markers of myocardial injury and ventricular stretch/strain, respectively, they carry significant limitations as markers of cardiotoxicity. Both markers lack standardization of measurement assays across centres, which can confound potentially clinically significant changes [69]. Furthermore, though sensitive for any injury or strain to cardiomyocytes, their specificity for CTRCD is limited as numerous medical conditions including ischaemic heart disease, cardiac arrhythmias, pulmonary emboli and sepsis can cause elevations in these markers. While these markers can be elevated in the acute setting during or immediately after treatment, numerous studies have highlighted their poor predictive value in monitoring for late-onset cardiotoxicity, a common manifestation from treatment with anthracyclines or HER2 inhibitors [70].

### Imaging modalities

While alterations in cardiac biomarkers reflect structural myocardial changes in the form of cardiac injury or strain, cardiac imaging provides a new avenue for surveillance of CTRCD as a functional assessment of the heart. Transthoracic echocardiography (TTE) is a readily available ultrasound imaging tool that can provide a comprehensive structural and functional assessment of the chambers of the heart, its valves, and the surrounding vasculature. It provides standardized measurements that are relatively consistent across countries and frequently updated by numerous expert cardiac bodies throughout America, Europe, and Asia [71–73]. It is the most commonly used modality for monitoring for cancer therapy-related cardiotoxicity, with most major bodies defining CTRCD as a reduction in LVEF of > 10% from baseline, to a value less than 53% [74]. LVEF is a critical marker of overall cardiac health and one of the best predictors of cardiovascular outcomes across all patient groups [75]. However, it has limited sensitivity, with reductions in LVEF often a late manifestation of cardiotoxicity and may reflect a missed opportunity for early intervention [76]. Further limiting its sensitivity, reductions in LVEF where it remains > 45% are not associated with increased mortality [77]. Other limitations include its significant inter-observer variability between operators and its lack of assessment of left ventricular diastolic dysfunction, which can precede systolic dysfunction in breast cancer patients treated with cardiotoxic chemotherapy [78, 79].

Measurement of strain by speckle tracking echocardiography is a relatively new technique that provides a new method of assessment of left ventricular function. While LVEF relies on changes in LV volumes in systole and diastole, myocardial strain measures systolic function by changes in tissue deformation across multiple segments of the LV, averaged to obtain a single result often denoted as a negative value (representing myocardial fibre shortening in systole) [80]. LV global longitudinal strain (GLS) reflects the longitudinal contractility of the LV myocardial fibres and has been shown to have better intra- and inter-observer variability compared to LVEF [81]. It is a more sensitive marker of subclinical systolic dysfunction, with reduction in LV GLS noted prior to overt changes in LVEF, and allows risk stratification in those with LVEF > 45% [77]. It has also been demonstrated as a superior predictor of cardiovascular outcomes and all-cause mortality compared to LVEF [81]. In cancer patients treated with cardiotoxic chemotherapy, reduced baseline LV GLS demonstrated significant predictive value for the development of cardiotoxicity during treatment and demonstrated utility in detection of subclinical LV systolic dysfunction on follow-up TTEs [80]. While some studies have shown benefits of early intervention with cardioprotective therapy in those patients with ‘subclinical’ cardiac dysfunction, further research is required to validate these results [82]. However, this is now a class I recommendation by the European Society of Cardiology (ESC) to be performed at baseline and follow-up of patients at risk of cancer therapy-related cardiotoxicity [83]. Given the risk of late-onset cardiotoxicity, particularly with anthracyclines, some studies recommend longer 5- and 10-year follow-up TTE in patients at higher risk, including those who received higher cumulative doses of anthracyclines, those over 60 years of age at the time of treatment, and those with baseline cardiovascular risk factors [84]. The ESC has now incorporated LV GLS as a marker of CTRCD, with a reduction in LV GLS > 15% from baseline even in the presence of normal LVEF representative of mild CTRCD. However, LV GLS is load-dependent and thus its accuracy is limited in haemodynamic overload [85]. Furthermore, measuring LV GLS requires acquiring images at high frame rates, requires an operator experienced with strain imaging, and has limited accuracy with poor image quality [86]. Similar to LVEF, it does not reflect changes in diastolic function; with HFpEF, the more prevalent phenotype at long-term follow-up in breast cancer patients compared to HFrEF [87].

There are several novel echocardiographic parameters that are being explored as potential markers of early cardiotoxicity. LV global circumferential strain (GCS) and right ventricular free wall strain (RV FWS) measure LV torsional contraction and RV longitudinal contraction, respectively, both using speckle tracking echocardiography. They have been shown in studies to identify a greater number of cancer patients with subclinical cardiac dysfunction compared to traditional parameters [88]. Left atrial phasic strain (particularly reservoir and conduit strain) has also been investigated as markers of early or ‘subclinical’ diastolic dysfunction superior to traditional Doppler-derived parameters [89]. Reductions in left atrial strain have been identified in cancer patients treated with cardiotoxic therapies prior to changes in LVEF or symptoms, and have also been identified in a greater number of patients compared to those with significant reduction in LV GLS, consistent with prior studies highlighting diastolic dysfunction as an earlier marker of cardiotoxicity [32, 89]. Myocardial work, an echocardiographic measure derived from LV GLS and brachial systemic blood pressure, has also been demonstrated as a potential marker of early cardiotoxicity in cancer patients [90]. Studies have highlighted its sensitivity, potentially more so than LV GLS, and also its lack of influence from loading conditions. While most patients are typically monitored using TTE to measure LVEF and LV GLS, cardiac biomarkers are often utilised as an adjunct given their sensitivity to myocardial injury in high-risk patients with pre-existing cardiovascular disease [67].

Cardiac magnetic resonance imaging (CMRI) has emerged as a potential surveillance modality for cardiotoxicity. It provides the most accurate assessment of chamber structure and function, including strain, and can more reliably identify marginal changes in left ventricular function compared to traditional TTE. Beyond TTE, it can also better delineate between and characterise endocardial, myocardial, epicardial and pericardial tissue, identifying areas of oedema, inflammation or fibrosis to better understand the pathophysiology of cardiotoxicity [91]. However, CMRI is quite costly, time-consuming, and much less readily accessible when compared to TTE [92]. In addition, many patients are precluded from the scan due to the presence of metallic foreign bodies or implants. For these reasons, its use in clinical practice for surveillance of cardiotoxicity in cancer patients has been limited.

Surveillance for treatment-related cardiotoxicity in breast cancer patients is crucial in reducing morbidity and mortality. Through early recognition of ‘subclinical’ myocardial injury using more sensitive biomarkers and imaging tools, early cessation of cardiotoxic treatment, longer surveillance in select patients at higher risk of late-onset cardiotoxicity, and prompt initiation of cardioprotective medications can prevent irreversible myocardial injury and reduce mortality [33].

## Future directions and conclusion

Whilst many of the imaging markers have demonstrated either adequate sensitivity or specificity for the identification of myocardial injury, there has been difficulty achieving both. Future studies continue to investigate novel markers and advanced imaging techniques that might better predict patients at risk of developing CTRCD. Using a ‘multimodal’ approach combining imaging with biomarkers could be advocated for patients at high risk of developing CTRCD. However, further research is needed to develop standardized guidelines on surveillance and management of this vulnerable patient cohort.

Cardio-oncology is an emerging field involving multidisciplinary care between Oncologists and Cardiologists looking to prevent, monitor, and treat cardiotoxicity, while preventing interruption in delivery of chemotherapy in cancer patients. While this has been particularly developed in breast cancer patients who are often exposed to treatments that carry significant risk of cardiac damage, this collaborative approach of care should extend to other cancer patients who receive cardiotoxic chemotherapy.

## Abbreviations

BNP: B-type natriuretic peptide
CMRI: cardiac magnetic resonance imaging
CTRCD: cancer therapy-related cardiac dysfunction
EGFR: epidermal growth factor receptor
ER: estrogen receptor
ESC: European Society of Cardiology
GLS: global longitudinal strain
HER2: human epidermal growth factor receptor 2
HFpEF: heart failure with preserved ejection fraction
HFrEF: heart failure with reduced ejection fraction
LV: left ventricular
LVEF: left ventricular ejection fraction
NT-proBNP: *N*-terminal pro-B-type natriuretic peptide
PD-1: programmed cell death protein 1
PD-L1: programmed cell death ligand 1
PR: progesterone receptor
ROS: reactive oxygen species
TTE: transthoracic echocardiography

## Declarations

### Copyright

© The Author(s) 2026.
